# The Development of a Personalised Training Framework: Implementation of Emerging Technologies for Performance

**DOI:** 10.3390/jfmk4020025

**Published:** 2019-05-16

**Authors:** Craig Pickering, John Kiely

**Affiliations:** Institute of Coaching and Performance, School of Sport and Wellbeing, University of Central Lancashire, Preston PR1 2HE, UK

**Keywords:** genetics, metabolomics, cfDNA, miRNA, machine learning, pharmacogenomics, monitoring

## Abstract

Over the last decade, there has been considerable interest in the individualisation of athlete training, including the use of genetic information, alongside more advanced data capture and analysis techniques. Here, we explore the evidence for, and practical use of, a number of these emerging technologies, including the measurement and quantification of epigenetic changes, microbiome analysis and the use of cell-free DNA, along with data mining and machine learning. In doing so, we develop a theoretical model for the use of these technologies in an elite sport setting, allowing the coach to better answer six key questions: (1) To what training will my athlete best respond? (2) How well is my athlete adapting to training? (3) When should I change the training stimulus (i.e., has the athlete reached their adaptive ceiling for this training modality)? (4) How long will it take for a certain adaptation to occur? (5) How well is my athlete tolerating the current training load? (6) What load can my athlete handle today? Special consideration is given to whether such an individualised training framework will outperform current methods as well as the challenges in implementing this approach.

## 1. Introduction

Within recent years, there has been an increased interest in the ability to provide personalised—or at least more personalised—information to athletes, their coaches and support staff in order to enhance the athletic preparation process [1,2,3,4]. The sources of this information are potentially varied, but include genetic [1,2], epigenetic [1,2], workload-based [5] and wellness-based [6] information. Alongside this interest in personalised training, there has been a dramatic increase in the number of technologies available for both athletes and practitioners to utilise within the field, with varying degrees of validity [7]. Some of the results from early research in this area are promising [8,9,10]. However, many of these early findings require replication, and, at present, the research tends to be siloed; i.e., authors explore the impact of genetics on performance, as opposed to integrating it within either existing frameworks or alongside other emerging technologies, both of which would better mirror what happens within the field [11]. 

There is an increased recognition of the need to validate existing technologies [7], as well as to understand emerging trends in order to maintain a competitive advantage [12]. In seeking to enhance performance, researchers should aim to further bridge the gap between lab and field, focusing on the practical applications of their work, and support coaches and athletes in their quest to enhance performance [11]. Specifically, we need to better understand how we can use a variety of emerging technologies to better assist coaches in enhancing their athlete’s performance by getting closer to a definitive answer to the following questions.

(1) To what training will my athlete best respond?
(2) How well is my athlete adapting to training?
(3) When should I change the training stimulus (i.e., has the athlete reached their adaptive ceiling for this training modality)?
(4) How long will it take for a certain adaptation to occur?
(5) How well is my athlete tolerating the current training load?
(6) What load can my athlete handle today?

This review aims to explore novel methods which, when used alongside existing technologies, will hopefully help coaches gain answers to the above questions. These methods should assist in the decision-making process, allowing for the targeted use of emerging technology to guide such decisions and contributing to an enhanced understanding of the way in which each individual responds to—both in terms of adaptation and fatigue—exercise training. Furthermore, we examine the practical implementation of these technologies, some of which are highly invasive and potentially expensive, allowing interested practitioners to analyse the cost: benefit ratio of each technology. 

## 2. A Personalised Medicine Approach to Performance?

The announcement of the Human Genome Project (HGP) led to the belief that we would soon understand the genetic and molecular underpinnings of disease, and, in turn, be able to develop personalised treatments for individuals to combat such diseases. This, coupled with the decreasing costs associated with genome sequencing, led the US National Human Genome Research Institute to formalise a 20-year plan aiming to translate the insights, from both the HGP and early pilot studies, into medical breakthroughs [13,14]. The spotlight was further shone on the promise of precision medicine by President Barack Obama, who, in his 2015 State of the Union address, proposed a vision for a Precision Medicine Initiative within the US [15,16].

The precision medicine movement has had some limited successes. For example, an enhanced understanding of the genetic mutations within CFTR which cause Cystic Fibrosis (CF) has improved treatment for many sufferers. Here, patients can now be stratified into subgroups based on their CFTR genotype; the mutation type determines the effectiveness of the drug ivacaftor in the treatment of CF. In the ~85% of patients expected to see a reduced effectiveness of ivacaftor, a second drug, lumacaftor, can be given in combination, which appears to enhance treatment effectiveness [16,17]. Similarly, it is understood that genetic variants help explain susceptibility to diseases [18,19] allowing for more personalised, targeted advice to be given to those with the increased risk [20].

Alongside disease prediction and management, an understanding of genetic variation has been used to personalise drug treatments through the field of pharmacogenomics [21]. Here, information on genetic variants known to influence drug pharmacokinetics or pharmacodynamics is utilised to guide drug selection and dosage [21], demonstrated by the success seen in genotyping both VKORC1 and CYP2C9 to optimise the dose of warfarin [22]. This information can also be used to prevent adverse drug reactions [23,24]; for example, variation in CYP2D6 leads to increased codeine sensitivity, requiring an alternative drug to be used [16]. Additionally, within the oncology sphere, there is the potential to sequence individual patient tumours, and then utilise this information to guide treatment options [25,26], such as the provision of larotrectinib in TRK fusion-positive tumours [27]. Alongside genomics and pharmacogenomics, precision medicine has expanded to utilise other “-omes” and “-omics” technologies [28,29], such as an understanding of the microbiome in human health and disease, epigenetics, transcriptomics, proteomics and metabolomics [22,29].

Despite the potential promise of precision medicine, the approach has yet to fully reach its potential, and has been subject to a range of criticisms regarding its effectiveness [30,31,32,33]. Nevertheless, it remains a tantalizing proposition for the integrated health management of patients, and, as research progresses and challenges are overcome, will hopefully assist in the prevention and treatment of a number of diseases [16]. Additionally, the precision medicine framework has been proposed as a future method to improve both health and performance in athletes [4]. In this case, both genetics and genomics, in partnership with additional -omic technologies, could be used to detect underlying conditions that may alter athlete health, such as Hypertophic Cardiomyopathy (HCM) [34], injury risk (e.g., COL5A1 [35]), exercise adaptation [1], nutritional requirements [36,37] and ergogenic aid use [2]. 

In the upcoming sections, we will detail how some of the methods inherent within the personalised/precision medicine process may be utilised within the elite sports sphere in the future, allowing for the development of the personalised training process.

## 3. The Use of Genetic Information within the Personalised Training Process

Exercise genetics research has grown over the last thirty years, from the seminal HERITAGE study [38,39,40] to Genome-Wide Association Study approaches [41,42], and, most recently, the development of Total Genotype Scores to utilise genetic information within the contexts of training programme design [8,9]. The use of genetic information within sport is currently contentious; with the present consensus from scientists being that it lacks sufficient evidence for its use, and is ethically problematic [43,44,45]. However, high-level athletes and support staff appear interested in utilising genetic information to enhance their performance, with recent surveys suggesting that ~10% of athletes have done so already, with many interested in doing so in the future [46]. 

The main ethical and scientific hurdle regarding the use of genetic testing is that it is ineffective as a means of talent identification [43,44,45]. Certainly, there is no evidence that it is effective in identifying talented young performers [43], with a number of hurdles required to be overcome before it can even be considered for use in this way. Nevertheless, there is emerging evidence to suggest that genetic information may hold utility within the athlete preparation process [1], providing information on exercise response [8,9], recovery speed [47,48,49,50] and injury risk [51,52]. At present, the influence of a small number of genetic variants on athletic preparation has been explored. An example is that of ACTN3, in which a common polymorphism (rs1815739) results in the transformation of an arginine base (R) to a premature stop codon (X) [53]. The stop codon prevents the protein from being produced, and, as a result XX genotypes have lower α-actinin-3 levels [53]. This is not associated with any disease state [53], but is associated with a lower fast-twitch muscle fibre percentage [54] as well as changes in muscle morphology [55]. While the R allele of this polymorphism is robustly associated with elite speed–power athlete status [56,57,58,59], because of the high prevalence within the general population (~82% of Caucasians possess it [60]) it is ineffective as a talent identification tool [43,61]. Due to its influence on muscle morphology, there is the potential for R allele carriers to demonstrate greater improvements following high-load resistance training [62,63], along with an apparent protection from damage induced via eccentric loading [62,63] and sports injury [62,63]. ACTN3 is also potentially implicated in bone remodelling [64], so there is the potential that this information could be used in the prediction and management of these injuries. 

The challenge within the exercise genetics sphere is a transition from observational and association-based information to evidence-led interventions. At present, the vast majority of research is focused on explaining the differences between two groups of performers, either in terms of elite athlete status or differences in exercise adaptation in response to a training stimulus [9,38,65]. While this information holds some utility, its usefulness is, arguably, limited; a first-team player at an elite sporting club does not require a genetic test for talent identification (and such a test likely would not be valid anyway), and their coaches have likely already experienced the heterogeneity in response to an exercise stimulus. Instead, there needs to be somewhat of a paradigm shift in exercise genetics research, enabling us to better understand how we can use this information to enhance training programme design. For example, while there is a large body of research demonstrating that both ACTN3 and ACE modify the response to strength training [65,66], there are far fewer studies exploring how to utilise this information to enhance the response of different genotypes. A theoretical paper [67] first explored this, suggesting that ACTN3 R allele carriers—those expected to demonstrate the greatest response to high-load resistance training—should prioritise high-load resistance training, with an emphasis on eccentric loading along with high-intensity interval training (HIIT). Conversely, those with the XX genotype were suggested to be better placed to undertake low-load, high-volume resistance training, minimising eccentric loading (to which they have an increased susceptibility for muscle damage [62,63]) and undertaking longer, lower-intensity aerobic training. 

To our knowledge, the first study to attempt to directly test whether genetic information may assist in training programme design was published in 2016 [8]. Here, subjects underwent genetic testing to establish their genotypes for 15 separate single-nucleotide polymorphisms (SNPs) thought to influence the adaptive response to resistance training. Using a total genotype score (TGS) approach, the subjects’ genotypes at each SNP were given a score between 0 and 4, allowing the calculation of whether they would be expected to respond better to “power-biased” (high-load, low-volume) or “endurance-biased “(moderate-load, high-volume) resistance training. The subjects were then randomly assigned to receive either genetically matched or mismatched training. The results showed that those undertaking genetically matched training—i.e., power-biased subjects undertaking power-biased training or endurance-biased subjects undertaking endurance-biased training—achieved around three times the magnitude of performance improvement in countermovement jump (CMJ) height and Aero3 tests. The results of this study suggested, for the first time, that genetic information could be used to enhance training adaptations. These findings were somewhat polarizing within the scientific community, receiving both criticism [68]—which was rebutted [69]—and praise [70]. 

Taken together, the results of this earlier work, both theoretical [67] and applied [8], suggest that we are at the start of the journey towards being able to utilise genetic information to enhance elite sport performance. How this information integrates with other -omes and other emerging technologies is explored further below.

## 4. Novel Markers of Exercise Adaptation and Recovery

### 4.1. Epigenetic Modifications—Novel Markers of Exercise Adaptation and Fatigue

Epigenetic modifications act to regulate genetic expression, and epigenetics can be very broadly defined as changes in genetic expression that occur without a change in the underlying genetic code. There are numerous different epigenetic changes that can occur, of which three are most well studied; DNA methylation, histone modifications and microRNAs (miRNA) [71,72,73,74]. Epigenetic modifications have the potential to be heritable [75], but also may be both malleable and transient [75], and have been proposed as potentially important modifiers of exercise adaptation [76,77].

#### 4.1.1. Methylation

DNA methylation refers to the addition of a methyl (-CH3) group to a cytosine (C) DNA base. The methyl group reduces the availability of the cytosine base to the DNA transcription machinery, which therefore limits the transcription of that particular section of the gene. Whether this is positive or negative is context-specific, depending on whether transcription of that specific gene is desired. For example, methylation of PPARGC1A, a gene involved in mitochondrial biogenesis, is associated with an increased risk of type II diabetes [78]. Conversely, methylation of a number of cancer promotor genes is likely positive, reducing the risk of the disease [75]. Regular exercise is able to both methylate disease-associated genetic variants and demethylate (i.e., remove the methyl group) genes associated with positive exercise adaptations [72,75,79]. This relationship is fluid and transient, with methyl markers associated with inactivity removed when the subject undertakes exercise training [72]. Recently, Seaborne and colleagues [80] demonstrated that skeletal muscle has an epigenetic memory with acute exercise producing methylation patterns that are maintained through a period of inactivity, which appear to subsequently enhance later adaptations to resistance training. 

As such, there is the possibility of utilising methylation patterns as markers of current status, providing insight to the training history of athletes. As research in this area develops, we might gain an understanding to what the implications of specific methylation patterns are, such that we could use this information to determine the responsiveness of a given athlete to a stimulus. Additionally, aberrant methylation patterns could be identified, and training programmes designed to remove those patterns, potentially enhancing subsequent exercise adaptation. 

#### 4.1.2. Histone Modifications

DNA is wrapped around structural proteins called histones, giving it a tightly coiled structure. The tightness of these coils makes the individual bases poorly accessible to the various different transcription factors and enzymes requires to transform the raw code of DNA to the required protein. To combat this, the body has evolved a method for various different stimuli—including exercise—to better access its DNA when required; that of histone modifications. Here, the histone proteins are acted on to allow the DNA to uncoil, making it more accessible for translation to the required protein. This primarily occurs via the addition of an acetyl group to the histone protein, which is catalysed by the histone acetyltransferase (HAT) enzyme group [81]. In turn, the acetyl group is removed by histone deacetylase (HDAC) [81]. 

Given their fundamental role to play to gene transcription, and, given that transcription of genes is a crucial aspect of exercise adaptation [82], it is clear that both HATs and HDACs have the potential to modify the response to exercise. This has been studied in mice models, where an increase in a specific HDAC—HDAC5—blunted the expected increase in type-I fibres following aerobic training [83]. Other studies have demonstrated how concentrations of both HATs and HDACs may alter muscle plasticity; acetylation of the histone H3, for example, has been linked to alterations in the expression of myosin heavy chain genes, which in turn potentially alters muscle fibre type [84]. As a result, the monitoring of HAT/HDAC concentrations may assist in understanding the training response. If there is an increase in those HATs/HDACs associated with an increase in type-I fibre following training, and the athlete is a sprinter, it would seem logical to modify the training stimulus to instead provide a more optimal adaptation. 

Of the three major epigenetic modifications, histone modifications are perhaps the least well understood, in part due to the fact that they are highly site-specific, so changes occurring within the muscle would require a biopsy. Given the largely transient nature of histone modifications, frequent biopsies would likely be required, a process which often is not feasible, especially in elite athlete cohorts, potentially limiting the application of this technology in practice. 

#### 4.1.3. miRNA

Traditionally, it was believed that RNA served as an intermediate step between DNA and the proteins produced; in this early model, RNA in the form of messenger RNA (mRNA) was produced from DNA during transcription, with the mRNA then being transported to the ribosome for production of the required protein. However, the results of ENCODE (Encyclopedia of DNA Elements) suggested that, while ~75% of our genome is transcribed into RNA, only a very small proportion (~3%) is directly involved in the creation of proteins [85]. This suggests that the vast majority of RNA is not involved in the creation of protein, but instead, in some cases, may alter the translation of proteins by controlling mRNA [76,86]. miRNAs play a role in modulating metabolism and inflammation, which in turn may modify exercise recovery and adaptation [76]. As such, they represent potentially important biomarkers in the personalised training process. 

The role of miRNAs in adaptation to resistance training has been explored in a few studies. Two miRNAs—miR-1 and miR-133a—are expressed during skeletal muscle hypertrophy [87]. Importantly, differences in miRNA concentrations may be able to predict exercise training response. Davidsen and colleagues [88] reported that high- and low-responders to a resistance training programme differentially expressed four miRNAs, with three (miR-378, miR-29a and miR-26a) downregulated in low responders and one (miR-451) upregulated in low responders. D’Souza and colleagues [89] reported that the signature of five miRs could distinguish between powerlifters and healthy controls with 100% accuracy. Similarly, Horak et al. [90] demonstrated that baseline levels of miR-93 represented an independent predictor of improvements in isometric leg extension following a resistance training programme. 

miRNAs have also been implicated in modifying the response to aerobic endurance training. Nielsen and colleagues [91] demonstrated that specific miRNA concentrations varied in response to both an acute aerobic training session, as well as a longer-term (12-week) training programme, a result which has been replicated [92]. Aoi and colleagues [93] demonstrated that a specific miRNA, miR-486, was significantly decreased following both acute and chronic endurance training when compared to baseline, and the ratio of this change was negatively correlated with changes in VO2max. Additionally, Domanska-Senderowska et al. [94] found a correlation between miR-29a and VO2max training improvements in a group of soccer players. miRNAs may also be useful in assessing baseline fitness, with three (miR-210, miR-21 and miR-222) associated with lower VO2max [95]. 

Furthermore, the manipulation of training variables has been demonstrated to affect miRNA levels. As an example, Schmitz and colleagues [96] reported that 4 x 30s high-intensity sprint running repetitions significantly increased both miR-222 and miR-29c levels, while 8 × 15 second sprints did not. Both of these miRNAs are associated with adaptations to exercise; miR-222 plays a role in exercise-induced cardiac growth [97], while miR-29c is a modulator of cardiac muscle remodelling [98]. Additionally, miRNAs appear, at least in some cases, to be sensitive to exercise dose, plateauing if there is insufficient progression [99]. 

miRNAs also hold potential as markers of exercise load. As an example, Gomes and colleagues [100] reported that three miRNAs (miR-1, miR-133a and miR-206) were significantly elevated following a half-marathon when compared to baseline. Further research examined the differences in miRNA release following 10 km, half-marathon and marathon runs [101], with the extent of miRNA increases distinct between the distances. These specific miRNAs were associated with inflammation, suggesting that athletes and practitioners may be able to better quantify and understand the individual inflammatory response to exercise, allowing for more personalised recovery processes to be put in place. Recently, Hakansson and colleagues [77] identified differences in miR-29a-3p (which was also identified by de Gonzalo-Calvo et al. [101]) and miR-495-3p expression between elite athletes and peripheral artery disease patients following exercise, suggesting that these miRNAs may hold promise as markers of muscle recovery following exercise. 

As such, the evidence suggests that the monitoring of miRNA concentrations, both before and during an exercise programme, may hold utility. The measurement of concentrations prior to beginning an exercise programme may be able to identify high- and low-responders to that intervention [88,90], allowing for the modification of the subsequent training programme. Similarly, the monitoring of miRNA concentrations during the training programme may act as a real-time monitor of adaptation, with increases or decreases in specific miRNAs associated with a particular training response [96]. In time, as research in this field progresses, it may be possible to match specific miRNAs to a specific molecular process; here, coaches will be able to understand whether the desired training effects—such as an increase in mitochondrial biogenesis—are actually occurring. We are close to the starting line here; the identification of miR-222 and -29c as drivers of cardiac adaptations following exercise illuminates the potential utility of monitoring the concentrations of these miRNAs—should an individual not see an elevation in these miRNAs, then training intensity/duration may have to be modified to elicit such a change [96]. Additionally, lower concentrations of miR-33 are associated with greater activation of AMPK following aerobic training [102], and miR-29b alters PGC-1α production [103]—both molecular signals for mitochondrial biogenesis—again demonstrating how real-time monitoring of miRNA concentrations could allow coaches to understand the specific adaptations an exercise is stimulating. Regular monitoring of miRNAs also has the potential to act as a marker of adaptation, as increases in specific miRNAs appear to be blunted when exercise dose is not progressed within a training programme [99]. Taken together, the evidence suggests that miRNAs have the potential to be utilised as biomarkers of training response [104,105], both in terms of adaptation and recovery. However, at present, one major limitation to the use of miRNAs as biomarkers is a lack of uniformity in response across studies; very rarely has a single miRNA been shown to have a universal response to a type of exercise [106]. For example, while increases in miR-1 and miR-133a have been shown following endurance exercise [107,108,109], other studies have found no such increase [101]. Further research will need to elucidate whether the miRNA response to exercise is heterogeneous (and potentially caused by heterogeneity in subjects [106])—limiting their use as exercise biomarkers—or if some commonalities can be found. 

#### 4.1.4. Utilisation of Epigenetic Markers within Training Programmes

As discussed above, the three major epigenetic modifications hold potential utility for a role within the personalised training process. Of these, perhaps the most promising are miRNAs, which have the potential to serve as markers of responsiveness to a training programme prior to that programme being undertaken [88], allowing for the coach to match the athlete to the required exercise type. miRNAs also hold potential value as a real-time marker of exercise adaptation [94], allowing for a change of stimulus to be applied at the most optimal time point, and as a marker of fatigue status [77], allowing for daily changes in training load and volume. Methylation markers have the potential to act as markers for previous training exposure [80], as well as giving guidance as to the current adaptive potential of an athlete at a given time [110], allowing for the required stimulus to be provided to the athlete. Finally, histone modifications may serve to allow the coach to better understand which stimulus provides which adaptive signals within each individual athlete, again allowing for a highly targeted approach to sports training. 

#### 4.1.5. Practical Perspectives

Perhaps the biggest issue facing the provision of epigenetic modifications within an exercise training context is that such changes are often both tissue specific and transient [111]. As a result, the accurate determination of epigenetic changes requires the sampling of the specific tissue, such as skeletal muscle, which can be both invasive and traumatic, and hence not palatable to high-level athletes. Additionally, the samples would have to be taken immediately after exercise for accurate analysis to occur. As epigenetic modifications can be both fast acting and temporary, frequent testing for such modifications would likely have to occur, increasing the cost and reducing the practicality of such technology. 

However, the collection of saliva for the profiling of DNA methylation holds promise [112], with methylation sites in saliva concordant with methylation within the target tissue for some specific biomarkers. To our knowledge, this has yet to be explored within an exercise setting but, if salivary DNA methylation profiling for exercise-related modifications becomes feasible, it will remove a substantial barrier to entry for methylation profiling within elite sport. 

#### 4.1.6. Section Summary

**Figure 1:**
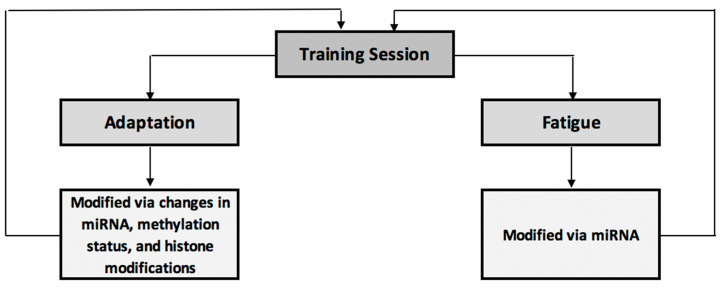
Summary of epigenetic influences on training-induced adaptation and fatigue.

Figure 1 acts as a brief summary of the impact of epigenetic modifications on exercise adaptations and fatigue. Here, an exercise training session elicits adaptive and fatigue-inducing effects, both of which are partially controlled via epigenetic modifications. These epigenetic modifications in turn have a feed-forward effect to the next training session, modifying performance, adaptation and fatigue response to that session.

### 4.2. Cell-Free DNA (cfDNA): A Novel Marker of Exercise Adaptation?

Circulating cell-free DNA (cfDNA) refers to the presence of DNA fragments within the blood [113]. At rest, small amounts of cfDNA are present in the blood stream; these concentrations have been demonstrated to increase under both acute and chronic physiological stress, such as sepsis, trauma, cancer and myocardial infarction [114]. This is also true of exercise. For example, following a half-marathon race, mean cfDNA concentrations increased from 18 pg/µL (baseline) to 335 pg/µL [115], with similar results being demonstrated following an ultramarathon [116]. This is also true for resistance training, where increases in cfDNA have been demonstrated following a single training session [117] and within a 12-week training programme [118]. 

While the mechanism underpinning the increased release of cfDNA during exercise is poorly understood, it appears that cfDNA is primarily released from cells involved in immune function [119]. The magnitude of cfDNA concentration increases also appears proportional to both exercise intensity and duration [119,120], and the time course of these changes is remarkably transient, with cfDNA concentrations often returning to baseline within 24 h, even after highly exhaustive exercise [115]. As a result, cfDNA represents a potentially novel biomarker for fatigue and recovery in exercise [113,119,120]. In subjects who undertook a 12-week resistance training intervention, cfDNA was strongly correlated with mean training load within each 3-week training sub-block [118]. The highest concentrations of cfDNA also corresponded to a decreased performance level, leading the authors to suggest that cfDNA was a potential biomarker for overtraining; such a finding is potentially crucial given that overtraining/unexplained underperformance syndrome is, at present, largely a diagnosis of exclusion [121]. Finally, Haller and colleagues [122] demonstrated that cfDNA increases were proportional to, and strongly correlated with, total running distance in a group of soccer players. The 23-fold increase in cfDNA concentrations demonstrated in this study is believed to be the largest biomarker increase reported following acute exercise training, suggesting a greater sensitivity than more traditional markers, such as lactate and CRP. Furthermore, the correlations between cfDNA and RPE are stronger (*r* = 0.58) than for that of lactate and RPE (*r* = 0.32), again demonstrating its potential utility as an exercise load biomarker [122]. 

The collection of samples for measurement of cfDNA is relatively straightforward, requiring a small amount of blood to be collected from a capillary [119], which can easily be achieved through a finger prick. As such, the use of cfDNA as a biomarker of exercise load, recovery and overtraining is highly promising, especially given the evidence suggesting that it is more sensitive than traditional biomarkers of training load [122]; as a result, its use could represent an enhancement of current practice.

## 5. The Microbiome, Exercise and Elite Performance

The human gut plays host to more than 100 trillion microorganisms [123], which are collectively termed the microbiota. The roles these microorganisms play are multifaceted, assisting in the digestion of food [124], along with the production of nutrients such as vitamin K2 [125], the neutralisation of pathogens and carcinogens [126] and regulation of the immune system [126]. More recently, research has shown that the microbiota influence neurotransmitters, such as dopamine, via the gut–brain axis [127,128]. Additionally, it has been suggested that the microbiota may assist in the control of both the inflammatory response and oxidative stress during endurance exercise [129].

Because the microbiome is modifiable by both diet and exercise [130], knowledge of the current composition of an individual’s microbiome holds promise in guiding interventions. Currently, such interactions are poorly understood; while we understand that specific dietary changes, such as an increase in protein [131] or carbohydrate [132], modify the microbiome, the effectiveness of specific changes through targeted interventions has not been extensively tested. Additionally, while we understand that diversity of the microbiota is important, with elite athletes tending to have increased diversity compared to nonathletes, and active subjects demonstrating an increased diversity compared to inactive subjects [130], we cannot, as yet, offer more in-depth advice than “be active”. However, as knowledge in this area increases, it appears feasible that regular monitoring of an athlete’s microbiome will be able to inform dietary interventions targeted at enhancing immunity, substrate use during exercise, neurotransmitter response—which may assist in stress management—and post-exercise recovery. Accordingly, this represents a promising aspect of the personalised medicine approach to performance management in elite athletes.

## 6. Pharmacogenomics—Personalised Sports Nutrition?

Pharmacogenomics refers to the identification of genetic variants that modify the effects of a given drug, most commonly through alterations in pharmacokinetics (such as the metabolisation of that drug) or pharmacodynamics (such as variation in the drug’s receptor) [21]. Caffeine, a popular and well-utilised ergogenic aid [133], with its effects confirmed at meta-analysis level across a board range of exercise modalities [134], represents a potential candidate for the use of pharmacogenomics within sport. Here, genetic variation in CYP1A2—the gene encoding for cytochrome P450 1A2—affects caffeine metabolisation speed [135]. The evidence suggests that individuals with the AA genotype at a specific SNP—rs762551—within this gene metabolise caffeine quicker than C allele carriers [136], an example of pharmacokinetics specific to sports nutrition. Additionally, variation in ADORA2A appears to modify the binding characteristics of caffeine to the adenosine-2a receptor, which in turn alters caffeine’s effects on downstream dopamine transmission [137]; an example of pharmacodynamics. 

Knowledge of the differences in CYP1A2 and ADORA2A genotype may inform pre-competition caffeine strategies [2]. For example, CYP1A2 AA genotypes appear to experience greater ergogenic effects following caffeine ingestion than C allele carriers [138,139]; indeed, CC genotypes may even find some doses of caffeine ergolytic [139]. Similarly, early research suggests that subjects with the TT genotype of ADORA2A experience enhanced ergogenic effects of caffeine [140]. These SNPs also have the potential to modify habitual caffeine use [141], along with both anxiety [142] and sleep disturbances [143] following caffeine ingestion, suggesting that knowledge of genotype may enhance the decision-making process [2].

While caffeine offers the best example of pharmacogenomics within sporting contexts, a recent review [144] demonstrated similar interindividual variation in response to extracellular buffering agent supplementation (e.g., sodium bicarbonate). The interindividual variation is partially determined by differences in MCT1 genotype. MCT1 encodes for monocarboxylate transporter 1, which influences lactate ion transport. As such, variation in this gene may impact the effectiveness of buffering agent supplementation. As research in this area evolves, it may be possible to identify those athletes expected to see a greater response to a particular supplement [37], as well as modifying dosage and timing of various ergogenic aids [145] as a means to enhance performance.

## 7. The Integration of Other “omes”

Alongside an understanding of the microbiome, genome and epigenome, along with the utility of other markers such as cfDNA to act as novel markers of exercise adaptation and readiness, there are a variety of other “omes”, including the transcriptome, proteome and metabalome, which may enhance the personalised medicine approach to elite athlete preparation. At present, these aspects are poorly studied within an exercise setting, partly due to the complex technology and sampling methods required and the vastness of all quantifiable aspects of each -ome. 

The proteome is the term used to describe all the proteins expressed by our genome [146]. Given that these proteins are the direct drivers of exercise adaptation, involved in, for example, skeletal muscle hypertrophy and mitochondrial biogenesis [147], understanding the extent of protein expression in response to exercise, along with an understanding of interindividual variation in the expression of a given protein in response to a specific stimulus, may assist in the matching of the athlete to the training programme best suited to the desired adaptation, along with their personal biology. At present, proteomic measurement can be extremely invasive, requiring a biopsy of the required tissue; this is problematic for muscles, causing trauma which may reduce exercise performance and increase this risk of infection, and is impossible (at present) for organs such as the heart [148]. As a result, the majority of the studies exploring the proteomic response to exercise are carried out in rats, hampering out ability to achieve clarity from their findings.

Transcriptomics refers to the examination of mRNA levels genome-wide [29], with these RNA levels in turn thought to act as a measure of genetic expression. Interestingly, there have been wide differences in measured mRNA expression within muscle between trained and untrained subjects in response to exercise [149,150], suggesting that transcriptomics may hold promise as a “predictor” of training outcomes. More recently, however, authors have suggested that the association between increased mRNA expression and increased gene expression may not be as strong as once thought [151], and, indeed, may be due to technical error or random biological variation [152]; as a result, transcriptomics may not be as useful as proteomics within the personalised medicine approach to athlete preparation. 

Metabolomics refers to the measurement of multiple small molecule types that are downstream products of biochemical reactions [29]. Within the muscle, such metabolites could give insight into the type and rate of fuel being utilised, allowing for a personalised approach to sports nutrition. As an example, Starnes and colleagues [153] reported significantly reduced alpha-tocopherol levels following exercise training in rats, suggesting that the maintenance of vitamin E levels around exercise may be important in attenuating post-exercise muscle damage. Metabolites linked to epigenetic modifications, such as folate in the case of methylation [154] could also be monitored; this is of importance given that lower levels of methylation are potentially advantageous following a hypertrophy-orientated training session [110], again allowing for targeted, personalised nutritional practices to be recommended. Similar to proteomics and transcriptomics, the measurement the metabolome is, at present, highly invasive [155], limiting its potential applications to inform training programme design. 

In summary, while proteomics, transcriptomics and metabolomics hold potential promise as monitoring tools within the personalised training process, at present there are significant difficulties in utilising these technologies, given the highly invasive sample collection procedures, along with a lack of research within sporting contexts. As research in this field progresses, and sample collection techniques simplify, such an approach may become more feasible.

## 8. The Use of Technology in the Personalised Training Process

The utilisation of various different technologies within sport has grown over the last twenty years, from simple global positioning systems able to determine distance covered [156] to implantable devices able to measure force and strain on a muscle or tendon [157]. The increased growth of technology has led to a number of recent reviews on the subject [7,158,159,160], with interest on using these technologies to design training that better matches competition performance [156,158], manage fatigue [160,161] and reduce injury prevalence [161], although the level of validation of these technologies is highly variable [7].

An in-depth overview of the various different technologies is beyond the scope of this paper, but it is worth considering how these various technologies could fit into the personalised training process. From the perspective of training programme design, real-time and retrospective data gained from these technologies can be used to design optimal programmes. For example, in the preparation of an elite sprinter, power, force and velocity profiles can be determined through the use of timing gates [162], force platforms [163], wearable technology (e.g., senor insoles [164]), smartphone apps [165], accelerometers embedded in external training aids [166] and high-speed video [167]. In recent times, many of these technologies have been integrated into Inertial Measurement Units (IMUs) (reducing the number of separate systems that require ongoing administrating) and streamlining the data management process [168,169]. The data collected allows the coach and support team to determine the athlete’s current strengths and weaknesses in relation to their preferred gold-standard model and/or competition performance data, with specific exercises developed to address these weaknesses [170]. Such an approach has been utilised to personalise optimal loading strategies in resisted sprint training [171]. Additionally, McGuigan and colleagues [158] discussed the use of a battery of strength tests to determine strength and weaknesses across the strength and power domain, again allowing for enhanced personalisation of the training process. 

Technologies can also be utilised to quantify training load, which is useful in assessing fatigue and readiness to train [172,173,174]. This occurs via the quantification of both external (e.g., running velocity, duration and intensity, and weightlifting sets, reps and weight) and internal (e.g., heart rate (HR) and heart rate variability (HRV)) loads, along with the determination of environmental aspects that might affect such loads, such as temperature and altitude [175,176]. This can also be the case in contact sports, where wearable technologies such as accelerometers may assist in the quantification of “contact load”, which in turn has its own recovery requirements [177]. This information can then be used to better understand whether the training load is sufficient to promote the required adaptations and protect against injury, or too great, increasing injury risk [5,177,178,179,180,181]. 

Technologies can also be used to assist the coach and practitioner in determining readiness to train. For example, the use of a pretraining countermovement jump (CMJ) or measurement of bar velocity can assist in understanding the neuromuscular fatigue status of the athlete prior to training [182,183,184], while metrics such as HR and HRV (measured via chest straps or smartphone apps) assist in understanding the athletes readiness to train [185,186,187]. 

Furthermore, technologies are becoming increasingly ubiquitous within the athlete’s non-training life, leading to the creation of the “24-hour athlete” [188]. This includes the assessment of sleep measures, including duration and quality [172], which, given the effects of poor sleep on performance [189], recovery [190], cognitive function [191] and overall health [192], is an important management metric. 

While it is easy to get carried away with the latest technology, it is worth keeping in mind that subjective markers of training load and athlete wellbeing, such as mood and perceived stress, have been shown to outperform a more high-tech approach [6], demonstrating that a more targeted use of technology, along with common subjective markers, may represent the best approach at present.

## 9. Prediction, Data Mining & Machine Learning

With an abundance of information available to the coach, recent research has focused on being able to better utilise this information to underpin decision making via prediction, either in terms of injury risk [193,194], postinjury recovery times [195], physiology (such as muscle fibre type [196]), training loads and fatigue [197,198], talent identification [199] and training plans [200,201]. These approaches utilise a variety of different statistical modelling techniques, including simple data analysis with a hold-out set for validation (e.g. [194]), more complex data mining techniques (e.g. [202]) and machine learning tools (e.g. [198]). 

As the predictive ability of these various models tends to increase with both the amount and quality of data inputs, such methods represent promise as part of the personalised training process. Predictive modelling has been used in medicine with some success. For example, using a relatively simple Genetic Risk Score (GRS) of just 13 single-nucleotide polymorphisms (SNPs), Ripatti and colleagues [203] were able to identify individuals with a 70% increased chance of developing coronary heart disease. In building on a single data type (i.e., genetic information) model, Khera and colleagues [204] recently developed a GRS algorithm utilising 6.6 million SNPs to identify individuals with a threefold increased risk of developing coronary artery disease. Similar single data input models have been utilised in sport. For example, Borisov and colleagues [196] utilised a 14 SNP model to predict muscle fibre type in 55 subjects, with a Receiver Operating Characteristic (ROC) of 81% for professional athletes, demonstrating strong concordance with muscle biopsies. Such a finding could be very useful within elite sport because muscle biopsy testing is highly invasive, limiting its use, while genetic testing is non-invasive. Similarly, Larruskain et al. [194] collected hamstring injury data over five seasons in an elite soccer team, along with genetic information. They then created a model of five SNPs, which demonstrated acceptable discriminatory ability to explain previous hamstring injury within that cohort. However, when applied to a hold-out data set used as validation, the model performed only as well as chance, demonstrating a lack of ability to predict injury. 

As a result of the Larruskain and colleagues [194] study, it is clear that the use of individual pieces of data is likely insufficient in the prediction of complex phenotypes and outcomes, such as injury, while it perhaps is sufficient for less complex phenotypes, as demonstrated by Borizov et al. [196], who used genetic information to predict muscle fibre type with success. Data mining refers to the conversion of raw data—such as that collected by the various technological and testing practices utilised in elite sport—to information which can then be analysed [202]. Machine learning focuses on the development of algorithms to analyse that information, with those algorithms adapting and correcting themselves as the number of inputs increases [205]. Both of these techniques have been utilised in medicine, with success in predicting heart attack risk and breast cancer survivability [206,207,208]. Within a sporting context, Vandewiele and colleagues [198] developed a machine learning model that predicted the session rating of perceived exertion (sRPE) of the whole training group, allowing the coach to understand the general training load of a prescribed training session before it occurs. Additionally, their model predicted sRPE of individual athletes prior to training, allowing for the tailoring of individual workloads and, with the addition of data collected within the training session (such as total distance covered), predict the post-training sRPE for individuals, allowing the coach to better understand the load of a given session and make changes to following sessions accordingly. This approach is potentially important, given difficulties in coaches and athletes accurately quantifying sRPE [209], and has the potential to enhance training adaptations and reduce injury risk. The model itself was reasonably complex, with the implementation of environmental data (e.g., temperature and humidity), individual characteristics (e.g., age, current fitness level, muscle fibre type and previous sRPE scores) and training statistics (e.g., distance, duration and heart rate zones). 

In summary, the use of various different models to predict a given outcome—such as injury risk, training load or fatigue—holds promise in sport; however, as of yet it has not been extensively studied. The quality of any predictive model depends on the ability to have effective informative inputs, with an emphasis on collecting reliable and valid data. Genotype remains a promising input to such models, having been utilised in both disease [204] and sporting [194] domains. The ability to record an increasing richness of information, such as epigenetic modifications, along with better quantification of present metrics, such as training load, should assist in the production of valuable predictive models in the future, which, with the application of machine learning, will constantly evolve to increase predictive power with the increasing amounts of data being entered into the model.

## 10. A Centralised Framework for the Development of a Personalised Training Process

Having identified a number of different emerging technologies that, if our understanding grows, hold potential in the development of the personalised training process, the next step is to understand their integration into a framework for their use.

Genetic variation exerts an influence on every aspect of elite athlete performance, including training adaptation [1], injury risk [51,52], ergogenic aid use [2], post-exercise recovery [47,48,49,50], athletic development [3] and, at some point, potentially the identification of elite athletes. Additionally, researchers have identified the effects of genetic variation on important aspects such as skill acquisition [210], psychological traits [211] and post-exercise fatigue [50], along with tangential factors that may impact athletic performance and preparation, such as nutrient requirements [212], microbiome composition [213] and bone health [214]. As such, we can conclude that genetic influences are a fundamental and consistent modifier of athletic preparation, the harnessing of which should enhance the preparation process. 

However, genetic variation does not exist in a vacuum, and indeed it is not the only aspect affecting athletic preparation. As such, it needs to be placed in the correct context; for any single SNP, the likely effect on a given outcome is often very small. The identification of large numbers of SNPs that affect a given trait, along with the creation of Total Genotype Scores (TGS) for that trait, will likely improve the predictive accuracy of genetic information. However genetics will only ever serve as part of the picture: it allows us to understand predisposition, which we can use to predict outcomes—and, as we have seen, serve as a useful, but incomplete, input to statistical models [194,196]—but the addition of additional pieces of information, explored within this paper, should enhance the personalisation process. 

**Figure 2:**
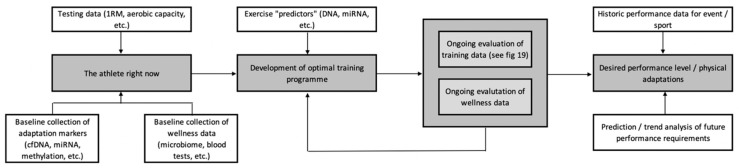
An overview of the development of the personalised training process.

Figure 2 serves as an overview example of how these various technologies might be integrated to enhance athlete preparation. When devising a training plan, we need to have a good idea of where we want to get to, i.e., what are the performance requirements of the athlete? This can be determined through the use of historic performance data, along with more complex predictions and trend analysis achievable through data mining and machine learning [215]. Once an understanding of the destination has been achieved, we need to know where the athlete is starting from. This can be achieved by collecting baseline fitness data, along with some of the adaptive markers discussed above (e.g., cfDNA), in conjunction with health and wellness data (e.g., microbiome). This information is then used, along with the integration of exercise “predictors” such as genetics [8,9] and miRNAs [216] to develop the optimal training programme, based around what the athlete is expected to adapt most favourably to. This plan should represent an initial outline, as opposed to a set prescription, given the highly variable nature of adaptation [217]—some, but not all, of which will be predictable from the information gained via the personalised training framework.

**Figure 3:**
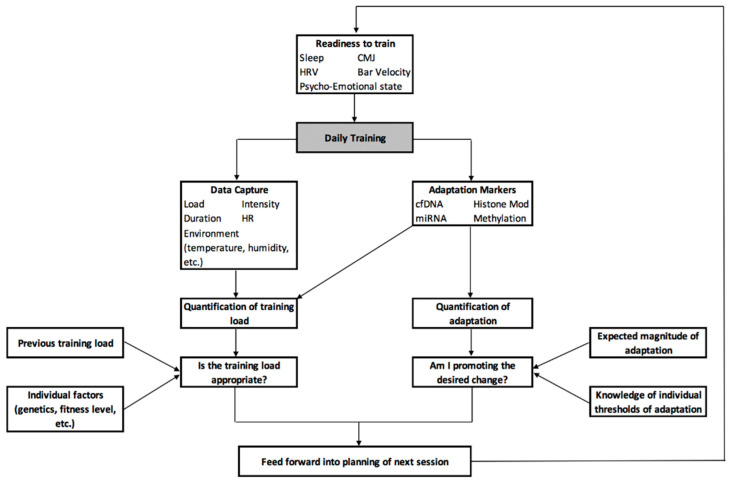
A framework for the implementation of various emerging technologies to enhance daily training practice.

Recognising this need for constant reformatting and reworking of the plan, Figure 3 provides an overview of how the various emerging technologies discussed in this paper can be used for the daily manipulation of training load, intensity and stimulus to meet the desired demands. Here, readiness to train and the adaptive state of the athlete is determined prior to training through the integration of metrics such as sleep, HRV, readiness testing (e.g., CMJ or bar velocity) and assessment of the psycho-emotional state, along with ongoing data on training load and current adaptive status determined from previous sessions. This information can then serve as an input to a statistical model that calculates the required training sRPE, similar to Vandewiele and colleagues [198] as detailed in Section 9. As training commences, data can be collected on aspects such as load, intensity, duration, heart rate response, environmental conditions, etc., and integrated to calculate the individual and team sRPE. Individual markers of adaptation and fatigue can then be collected from the athlete; cfDNA and miRNA can be used to assist in the quantification of fatigue and training load, with epigenetic markers used to establish whether the desired adaptations are occurring, and to what extent. Both aspects can then be compared to historical data, such as previous training load, and individual factors, such as genetics and fitness level, to understand whether the current training load is sufficient to promote adaptations, but not excessive enough as to increase the risk of injury. Similarly, in future it may be possible to use genetic information to determine a maximum threshold of possible adaptation, along with understanding what this adaptation looks like at the molecular level; this information can be compared to where the athlete is at a given point in time to determine if they have met this threshold—requiring a change in training goal—or if they can continue with the same training plan. 

Many additional technologies, both existing and novel, can be factored into these models as required. For example, blood testing for health markers, such as serum vitamin D, may be required; here, genetic variation can be used to predict the response to vitamin D supplementation [218] and to create individualised target reference values and nutrient requirements, an approach which has been highly successful in recent trials [219]. Microbiome sampling can occur less frequently, perhaps every three months, to monitor for changes. Pharmacogenomic principles can be utilised in the development of personalised sports nutrition strategies, such as the caffeine example described in Section 6, or to guide the selection of medications required to manage issues such as pain and trauma associated with daily, high level training and competition. 

With the variety of different information types that can serve for inputs into data models, data mining and analysis will be able to identify those with the largest effects on performance, adaptation, fatigue and injury risks, allowing for a more targeted approach to be taken to data collection if required. Furthermore, the integration of current technologies, such as urinary and salivary biomarkers [220], along with more standard physiological assessments, will likely enhance the predictive accuracy of these models.

The effectiveness of, and compliance to, a personalised training programme is currently unknown. In a pilot programme of 14 athletes, genetic information integrated into personalised injury prevention advice and techniques was found to reduce 12-month injury incidence, with more than half of the group finding the advice useful and implementing the recommendations [221]. The use of optimisation software to determine preseason training loads has proved successful in Australian Rules Football [222], while machine learning tools have been shown to outperform traditional methodologies in the prediction of response to training loads [223]. Furthermore, a personalised approach to training has demonstrated effectiveness, with individualised training based on force–velocity profiling [224] and HRV [225] shown to enhance training adaptations. A major challenge will be to get athletes to accurately and consistently collect data—such as sleep metrics and HRV—away from the training field, with ease of use and lack of perceived invasiveness important factors for technology developers to consider in this regard. Nevertheless, despite these challenges, the development of a personalised training process appears to hold promise in the optimisation of athlete performance.

## 11. Summary

Within this paper, we have explored the use of other novel technologies that, along with genetic information, may combine in the development of the personalised training process. While highly speculative and poorly researched, with the potential for significant practical issues, there is clearly scope for, and the acceptance of [46,221], an increasingly personalised training process as a method to enhance athlete performance. An important aspect of such a model is that athlete adaptation and performance is highly complex, with a number of biological systems interacting to create the outcome. The purpose of this paper is to demonstrate that, while genetic information likely does hold utility within elite athlete preparation, and potentially enhances the training process, it is crucial to keep in mind that genetic information represents only part of the picture. As research in this area grows, we will get a better understanding of how genetics influence elite athlete performance, as well as how we can integrate the information from genetic testing within a holistic athlete preparation model, which in turn will enable a more personalised approach to athlete preparation. This could become highly complex, with the collection of large swathes of data to act as an input for complex predictive models, but there is a simple message contained within this; that each athlete has their own unique biology, and every day presents in a slightly different state—adaptive or maladaptive—that requires the coach to make changes on the fly. The better informed the coach and athlete are, the better the decisions they can make, but, more importantly, we now understand that we cannot treat all athletes the same way, with one size fits all training programmes planned months in advance. Despite the potential complexity of a personalised training process, perhaps the simplest message—that all athletes need to be treated differently—is the most important.
